# Effects of a nurse-led multicomponent intervention for frail older adults living alone in a community: a quasi-experimental study

**DOI:** 10.1186/s12912-021-00801-1

**Published:** 2022-01-17

**Authors:** Mi Sook Song, Sunjoo Boo

**Affiliations:** Research Institute of Nursing Science, College of Nursing, Ajou University, 206 Worldcup-ro, Yeongtong-gu, 16499 Suwon, South Korea

**Keywords:** Aged, Depression, Exercise, Frailty, Physical functional performance, Quasi-experimental study, Social support

## Abstract

**Background:**

Given the rapid aging of the population in Korea, efforts to slow down or prevent frailty, to support the health of older adults, should be an important public health priority. This may allow them to continue living within the community by keeping their functional independence for as long as possible. This study aimed to evaluate the nurse-led multicomponent intervention for community-dwelling pre-frail or frail elderly on physical and psychosocial outcomes.

**Method:**

A non-equivalent control pre-, post-, and follow-up test design was used with a sample of 126 prefrail or frail older adults (62 in the experimental and 64 in the control group). The 12-week multicomponent intervention for the experimental group comprised physical exercise, cognitive training, and nutrition and disease management education. Outcome variables (Timed Up and Go Test results and measures of frailty, handgrip strength, depression, social activity, and social support) were measured both pre- and post-intervention, and after the 12-week follow-up period.

**Results:**

For each group, we assessed the significant interaction of time with frailty, depression, social activity, and social support, as well as Timed Up and Go Test results. In the experimental group, levels of depression decreased while levels of social support and social activity increased from each measurement period to the next, within the 12-month study period; those in the control group were relatively stable over time.

**Conclusions:**

The results indicate that nurse-led multicomponent intervention was effective for improving physical and psychosocial function of the (pre)frail older adults living alone in Korea, suggesting that older adults can take proactive roles in conducting their daily life and managing their health. A strategy for disseminating widely sustainable nurse-led multicomponent interventions should be developed for community-dwelling frail elderly who live alone.

## Background

The demographics of South Korea are rapidly shifting toward those of a super-aged population due to complex factors such as increased life expectancy, aging of the baby boomer generation, and declining fertility rates. The proportion of those aged 65 years or older rose from 7% to 2000 to 14% in 2017 and is expected to reach 20% by 2025 [1]. Moreover, the pace of population aging in Korea is faster than that of Western countries. While longevity can present opportunities for older adults, their families, and society, the extent of those opportunities depends largely on these individuals’ health.

Aging can be accompanied by declines in physical and cognitive function, which negatively affect health and independence. Frailty has several approaches and operationalizations [2]. Recently, frailty is designated as a multidimensional concept, which encompasses losses in physical, psychological, and social functioning, and increases vulnerability to adverse health outcomes such as disability, hospitalization, or death [3–6]. At present, approximately 9.2–20.2% of community-dwelling older adults are categorized as frail in South Korea [4, 7, 8]. Importantly, pre-frailty, an early and reversible state before the onset of established frailty, has higher prevalence among community-dwelling older adults, ranging from 38.0 to 71.6% [8–10]. Given the rapid aging of the Korean population, efforts to slow down or prevent frailty and to keep older adults healthy and prolong their ability to live within the community should be an important public health priority. It can improve the quality of life among older adults and reduce heavy socioeconomic burdens.

Importantly, the prevalence of frailty is higher in vulnerable older adults of comparatively lower socioeconomic status (SES) [7, 10]. In particular, socioeconomically vulnerable older adults living alone are more likely to face barriers in accessing health care resources and lack social support. Such vulnerabilities may lead to greater risks of frailty [11] and vice versa. In Korea, home-visiting nursing services in public health centers are designed to improve access to, and sustainability of, healthcare for socioeconomically vulnerable population. They are public health efforts at a national level by the Regional Public Health Act (Act No. 16,262, Article 11.5) [12]. The main target for the service is lower SES prefrail or frail older adults living alone.

Fortunately, frailty may be reversible with appropriate interventions [13]. Targeted approaches to preventing frailty, ideally before the onset of functional decline, have been proposed to decelerate the frailty process and maintain physical function among older adults. Since frailty is affected by multiple factors, it is highly appropriate to apply multidimensional interventions, targeting multiple contributing factors, than to address individual aspects. Several randomized controlled trials of such multidimensional interventions have reported beneficial effects on physical function, nutritional status, and depression among community-dwelling older people [14–16]. Physical exercise programs, which are the basis of most interventions targeting frailty, have been shown to confer consistent favorable effects on physical function and muscle strength. Meanwhile, cognitive training and nutritional support play assistive roles in maintaining physical function in older adults [17].

In Korea, community-based nurse-led interventions have focused more on individual-based case management [18, 19] than social group approaches. However, psychosocial outcomes are important for older adults with frailty, who live alone, because their psychosocial status affects their physical energy and motivation for self-care [1]. According to general consensus, comprehensive community-based intervention is beneficial to prevent disability. It increases physical and social health in community-dwelling frail older adults [20], although less information is available on the comprehensive impact of a nurse-led multicomponent intervention on frail community-dwelling Korean older adults who live alone. In this study, we aimed to evaluate a nurse-led multicomponent intervention targeting community-dwelling older people living alone with an increased risk of frailty. More specifically, the aim of the present study was to evaluate the nurse-led intervention on the following outcomes: (a) frailty, (b) dynamic balance ability (Timed Up and Go Test [TUG]) results, and (c) handgrip strength. Evaluated psychosocial outcomes include (b) depression, (b) social activity, and (c) social support.

## Methods

### Study design, setting, and sample

A non-equivalent, controlled, pre-, post-, and follow-up test design was employed to identify the effectiveness of the multicomponent interventions for pre-frail or frail older adults of low SES who live alone. The study comprised a 12-week intervention and a 12-week follow-up period from May to November 2019. Samples for this study were selected from those who were enrolled in a public health center as a candidate for home-visiting nursing services, in a city near the capital city of Seoul, Korea. The services primarily focus on socioeconomically vulnerable populations, thus low SES prefrail or frail older adults in the community were considered as the candidates of this study.

Potential participants were identified through community screening. Eligible participants were (1) aged 65 years or older, (2) living alone, and (3) classified as pre-frail or frail. Potential participants were approached by a trained research assistant and informed about the purpose of the research and its voluntary nature. Upon providing written informed consent, each participant underwent a baseline assessment. Frailty was screened using a comprehensive geriatric assessment questionnaire used in the Visiting Health Management Services of the Korean Ministry of Health and Welfare [12]. It consists of self-reported questionnaires and objective measures of complex mobility function. Self-reported items included questions about daily activities of living (five items: ability to use public transport, shop for small purchases, visit the bank, venture out, and attend counseling), mobility (five items: climbing stairs, standing from a chair, walking for 15 min, history of falling, and fear of falling), nutritional status (five items: weight loss and body mass index, chewing or swallowing difficulties, and having a dry mouth), social activity (two items: number of outings per week and the frequency compared to the previous year), cognitive function (three items: forgetfulness, ability to make phone calls, and ability to recall past events), mood (five items), sensory function (one item: vision and hearing), and comorbidity. This information was collected through personal interviews conducted by a trained research assistant. The complex mobility function was measured using TUG. Frailty levels were calculated based on the scoring guidelines. Two points were assigned to the presence of any comorbidity, and an abnormal TUG; one point was assigned to a negative answer to the other items, yielding a maximum possible score of 31. Individuals were classified as *robust* if the frailty score was 0–3, *pre-frail* for scores 4–12, and *frail* for scores 13–31. Based on these criteria, those with scores of 4 or more were included in this study [12].

**Fig. 1:**
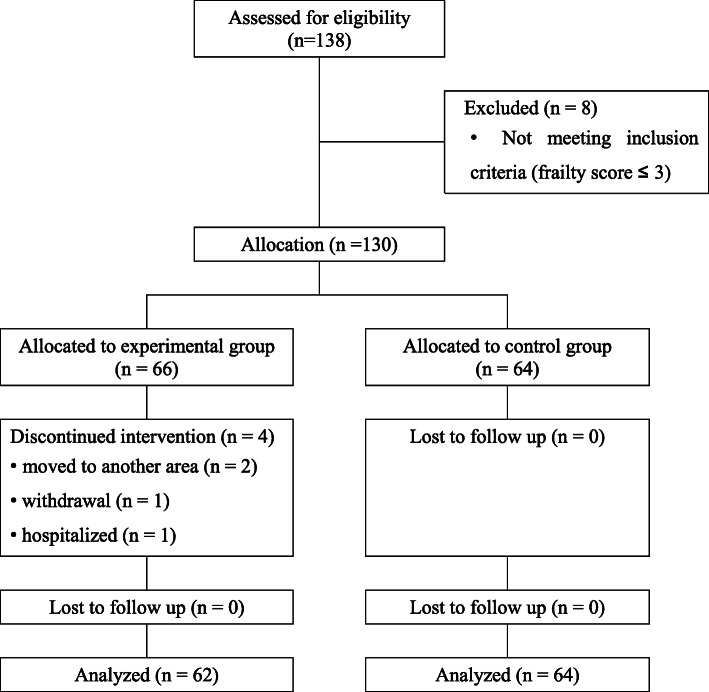
CONSORT flow diagram

The group assignment was based on the waiting list for home-visiting nursing services in the public center. According to the waiting list order, first the experimental group was assigned, and then the control group. A priori computation of sample size using G* Power version 3.1 revealed that 56 participants were required with an effect size (f) of 0.2, an alpha value of 0.05, and an actual power of 0.90. A total of 138 participants completed the initial assessments (Fig. 1). Among them, eight participants failed to meet the inclusion criteria (frailty score < 4), so 130 participants were assigned to the experimental (*n*=66) or the control (*n*=64) group. Four participants in the experimental group withdrew from the study. Therefore, the final sample of 126 participants (62 in the experimental and 64 in the control group) was analyzed in this study.

### Intervention

The 12-week multicomponent intervention for the experimental group was composed of exercise, cognitive training, and education for nutrition and disease management based on the multidimentional concept of frailty [2]. The intervention was conducted in accessible, attractive, and safe places, such as public health centers or senior centers to reduce the attrition rate. The intervention consisted of two 40-minute sessions once a week for 12 weeks. The first was a 40-minute group exercise session administered to approximately 10–15 participants by an exercise coach and trained exercise assistants. Each exercise session consisted of stretching (5 min), resistance exercises with elastic TheraBands (20 min), and aerobic movements (15 min) on rhythmic music selected by the participants to ensure a fun session. The intensity of the exercise was adjusted to suit the prefrail or frail elderly to reduce the follow-up loss. Each participant was trained for every activity according to their competence, allowing them to track their own exercise. They were closely monitored by the exercise coach and her assistants to prevent any injury. Resistance exercises focused on both the upper and lower extremities, emphasizing on muscles that are important for balance and gait control to prevent falls. The aerobic portion included stepping in standing positions or while sitting in a chair, as well as standing up from and sitting in a chair. Educational leaflets for each movement were distributed so that the participants could exercise at home even on days when they did not participate in the exercise class.

After the first exercise session, cognitive training session continued for another 40 min. The second session included either calendar making or Cup Nanta, alternatively every other week, to improve cognitive function and sociality. These facilitated reminiscence and attention through art activities and folk music performances. The calendar-making program was operated using various materials; participants were encouraged to draw fun memories or special events of each month individually, and seasonal landscapes. The calendars were decorated with various, easily accessible materials, such as aluminum foil, old clothes, dried grains, coarse salt, cotton ball, etc. This process not only enabled the participants to reminisce about their meaningful everyday lives but also, and more crucially, helped them fulfill a desire for education that may have remained unfulfilled during their childhood. It simultaneously enhanced their concentration and sense of accomplishment.

 Cup Nanta, a performance involving tapping cups on a desk according to a rhythm to achieve harmony, is designed to strengthen the fingers as well as to improve sociality and emotional bonds among the participants. The meticulous efforts taken by each member to produce a unified performance, by aligning the sequence and rhythm of each movement, and prevent errors, enhanced the overall performance.

Health education regarding nutrition and chronic disease management was provided once per month. Nutrition education or cooking classes focused on selecting healthy foods and convenient recipes. Physicians enhanced medication adherence and healthy lifestyle choices to the participants. They provided health education on therapeutic goals for managing chronic disease, blood glucose, blood pressure and self-management skills. In addition, therapeutic connections were formed between the participants and the medical doctors practicing in the participants’ area of residence for direct treatment and consultation, to enable continuous treatment and monitoring after the program intervention. Healthy snacks were provided to reduce the risk of attrition. To ensure the validity of the study’s results, the researchers who collected and analyzed the data did not participate in the program. They were blinded to the participants’ group assignments.

### Measures

Measures for the following outcome variables were completed three times: at pre-intervention, post-intervention, and at the 12-week follow-up.

#### Frailty

We used 28-item frailty index to evaluate the effect of the intervention in this study [12]. It contains questions (containing five items each) regarding instrumental activities of daily living, physical functioning, nutritional status, and cognitive functioning. Participants were asked to answer *yes* or *no* to each question. Subsequently, the scores for all items were summed to indicate the level of frailty. Possible scores range from 0 to 31, with higher scores indicating a higher likelihood of frailty.

#### Timed Up and Go test (TUG)

The complex mobility function was measured using TUG. Participants were asked to stand up from a standard armchair, walk 3 m straight, turn around, walk back, and sit down on the chair. The time from getting up from the seat to sitting again was measured in seconds.

#### Handgrip strength

The handgrip strength of the dominant hand was measured with a dynamometer (Tabita 6103) with participants standing upright, facing forward, with elbows fully extended and their feet shoulder-width apart. For each of the three measurement points (pre-intervention, post-intervention, and at follow-up), handgrip strength was measured in kilograms twice, with a one-minute interval, and the scores were averaged for the analyses.

#### Depression

The 15-item Geriatric Depression Scale-Short Form Korean Version (GDSSF-K) measured depression [21, 22]. Participants were asked to rate their mood status on a yes or no scale. The level of depression was expressed as the average of all item scores, ranging from 0 to 15. Higher scores represented a greater level of depressive mood. The reliability coefficient of the GDSSF-K in Kee’s study was 0.88 [22], and 0.92 in this study.

#### Social activity

A five-item social activity scale developed in a sample of Korean older women living alone [23] was used to assess the levels of social activity in this study. Participants were asked to rate the frequency of their social activity for particular purposes, such as friend gatherings and economic or religious activities, on a scale of 0 (not at all) to 5 (every day). Total scores were calculated by averaging item scores, with higher scores indicating a greater frequency of social activities. Cronbach’s alpha was 0.89 in the present study.

#### Social support

The 19-item Medical Outcomes Study Social Support Scale was used to measure the level of social support on a five-point Likert scale [24]. The total social support score was calculated based on the scoring guidelines, ranging from 0 to 100. Higher scores indicate higher levels of social support. The scale was found to have good internal reliability at the time of its development. In this study, the internal reliability was 0.94.

### Statistical analysis

Data were analyzed using IBM SPSS software (version 23.0; IBM Corp., Armonk, NY, USA). The variables were screened for potential errors, missing data, and outliers. Assumptions were checked for every statistical analysis. Descriptive statistics were calculated for all variables. Bivariate analyses (i.e., t-tests, chi-square tests, and Fisher’s exact test) were conducted to examine the homogeneity between the two groups. To evaluate the effect of the intervention over time, a mixed ANOVA (repeated measures) was used to compare the differences between the outcome measures of the two groups. Partial eta-squared values were presented as a measure of effect size. A value of 0.01 indicates a small effect size, 0.06 indicates a moderate effect size, and 0.14 indicates a large effect size [25]. Differences were considered statistically significant at *p* < 0.05.

## Results

### Characteristics of participants

A total of 126 older adults completed the study and were included in the analysis. Their mean age was 78.8 (± 5.39) years, and 90.5% (*n*=115) of the participants were women. Approximately 30% (*n*=37) perceived their health as good. The most common chronic disease among participants was hypertension (73.0%), followed by arthritis (67.5%) and diabetes (29.4%) (Table 1). There were no statistically significant differences in the study variables by group at the baseline (Table 2).

**Table 1:** Homogeneity of general characteristics (*N*=126)

Variables	Total	Experimental group (*n*= 62)	Control group (*n*= 64)	*p*
	n (%) or mean ± SD			
Sex (female)	114 (90.5)	56 (90.3)	58 (90.6)	0.954
Age	78.79 ± 5.39	79.56 ± 5.50	78.05 ± 5.21	0.114
Perceived good health	37 (29.4)	22 (35.5)	15 (23.4)	0.172
Hypertension (yes)	92 (73.0)	44 (71.0)	48 (75.0)	0.690
Diabetes (yes)	37 (29.4)	13 (21.0)	24 (37.5)	0.051
Stroke (yes)	5 (4.0)	3 (4.8)	2 (3.1)	0.677^*^
Heart disease (yes)	12 (9.5)	5 (8.1)	7 (10.9)	0.763
Cancer(yes)	12 (9.5)	6 (9.7)	6 (9.4)	1.000^*^
Arthritis(yes)	85 (67.5)	37 (59.7)	48 (75.0)	0.087
Incontinence(yes)	5 (4.0)	4 (6.5)	1 (1.6)	0.204^*^

^*^Fisher exact test

**Table 2:** Homogeneity of study variables (*N*=126)

Variables	Total	Experimental group (*n*= 62)	Control group (*n*= 64)	*p*
	Mean ± SD			
Frailty	10.23 ± 3.67	10.48 ± 3.84	9.98 ± 3.52	0.447
TUG (sec)	10.06 ± 5.95	10.47 ± 7.17	10.45 ± 7.69	0.989
Handgrip strength (kg)	19.60 ± 5.29	20.00 ± 5.23	19.22 ± 5.36	0.409
Depression	6.21 ± 3.18	5.87 ± 3.62	6.53 ± 2.67	0.247
Social activities	12.34 ± 3.67	12.97 ± 3.45	11.73 ± 3.79	0.059
Social support	44.12 ± 2.00	48.11 ± 22.82	40.26 ± 24.65	0.066

*Note.* TUG=Timed up & go test

### Effects of a nurse-led multicomponent intervention

The effects of the multicomponent intervention are summarized in Table 3. Repeated measures analysis showed a significant group-by-time interaction on the levels of frailty, TUG, depression, social activities, and social support. A relatively greater improvement was shown in the results reported from the pre-test to the post-test.

**Table 3:** Effects of intervention

Variables		Pre-test		Post-test		Follow-up test		source	*p*	*Partial Eta Squared*
		estimated mean (SE)								
Frailty	Exp.	10.48	(0.47)	8.53	(0.50)	8.40	(0.52)	G	.459	.004
	Cont.	9.98	(0.46)	9.33	(0.49)	9.52	(0.51)	T	<.001	.223
								G*T	.009	.073
TUG (sec)	Exp.	10.47	(0.94)	8.29	(0.94)	8.16	(0.96)	G	.212	.013
	Cont.	10.45	(0.93)	10.59	(0.92)	10.83	(0.95)	T	<.001	.191
								G*T	<.001	.270
Handgrip strength (kg)	Exp.	20.00	(0.67)	20.35	(0.64)	20.76	(0.68)	G	.217	.012
	Cont.	19.22	(0.66)	19.10	(0.63)	19.48	(0.67)	T	.060	.022
								G*T	.452	.006
Depression	Exp.	5.87	(0.40)	4.19	(0.44)	4.31	(0.44)	G	.012	.050
	Cont.	6.53	(0.40)	5.91	(0.43)	6.06	(0.43)	T	<.001	.201
								G*T	.036	.052
Social activities	Exp.	12.97	(0.46)	14.10	(0.46)	14.06	(0.50)	G	.002	.076
	Cont.	11.73	(0.45)	11.81	(0.46)	11.56	(0.49)	T	.006	.080
								G*T	.007	.077
Social support	Exp.	48.11	(3.02)	53.64	(2.98)	55.00	(2.99)	G	.005	.061
	Cont.	40.26	(2.97)	41.19	(2.94)	40.75	(2.95)	T	.001	.114
								G*T	.012	.070

*Note.* TUG=Timed up & go test; G=group; T=time

**Fig. 2:**
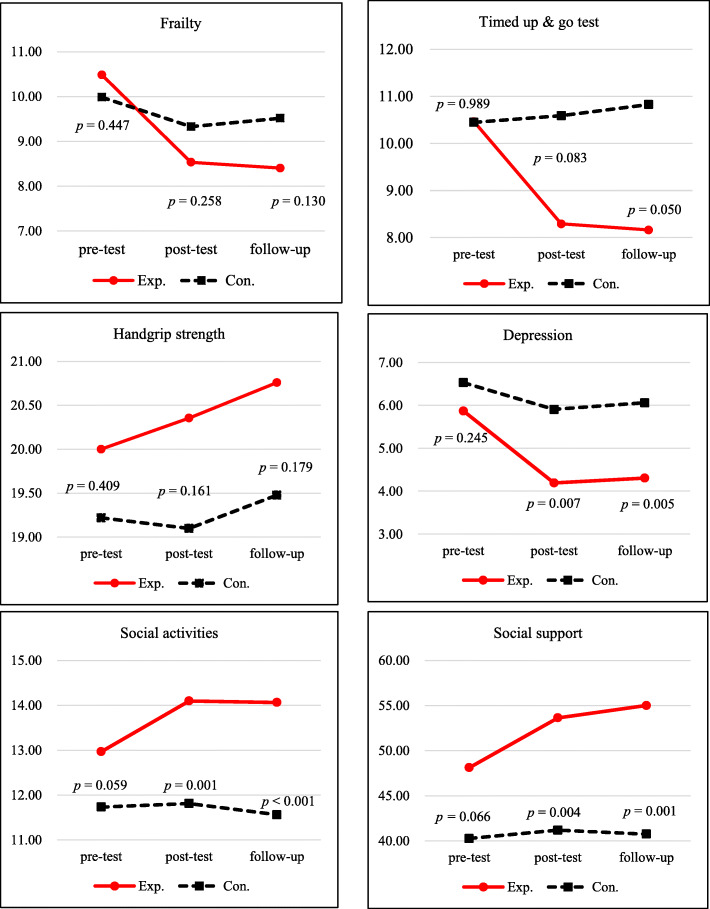
Effects of nurse-led multicomponent intervention

Post-hoc results showed a significant change in the levels of depression, social activity, and social support from pre-test to post-test (Fig. 2). The effects of the nurse-led multicomponent program in the experimental group were sustained over 12 weeks of follow-up.

## Discussion

Using a non-equivalent, controlled, pre-, post-, and follow-up test design, this study evaluated the effects of a 12-week nurse-led multicomponent intervention for pre-frail or frail older adults aged 65 or above. This study found significant interactions between group (experiment or control) and times for all outcome variables except handgrip strength. In the experimental group, depression, social support, and social activity continuously improved from the first measurement during the 6-month study period, while those in the control group were relatively stable over time.

Frailty includes impairments in physical, psychosocial, and cognitive functions. Thus, a multidimensional intervention that targets contributing factors of frailty could improve physical and psychosocial functions. Previous studies from other ethnic groups have reported that multicomponent interventions significantly improve participants’ frailty levels compared to single interventions [14, 16]. Regrettably, majority of the studies conducted in the Korean community settings have conducted a single intervention program such as exercise [26, 27], or have investigated the effects of an intervention for prefrail or frail adults merely on the basis of physical or psychological aspects [18, 26–28]. The studies did not consider or measure the impacts of socialization in individuals participating in the interventions. However, psychosocial components make an impact on physical energy and self-care motivation, which is particularly crucial for frail older adults who live alone [15].

Prefrail or frail older adults generally have low levels of social involvement, increasing their frailty [29, 30]. People living alone, especially, have a lower availability of personal assistance and greater social vulnerability [31]. Therefore, this study was conducted with an assumption that multicomponent intervention through the well-designed community social networks would be a solution to improve the health status of prefrail or frail older adults by activating their life pattern.

To facilitate socialization in this study, a small group rather than an individual-based approach was used. Levels of social activity and social support were measured to identify whether changes in the participants’ daily lives would produce improvements in the levels of frailty over the study period. Upon the intervention completion, a statistically significant improvement was found in the social activity and social support. The effects were sustained during the follow-up periods, which may lead to improvements in physical and psychological health, such as depression and frailty. We could explain that such positive changes in physical and psychological health, found in the experimental group, might be in part due to the intentional expansion of their daily activity spectrum through their regular participation in the comprehensive program across the 12 weeks, as they had previously lived alone and engaged in limited social activities. Therefore, our results contradict those of a previous study [32] that determined evidence regarding the effect of resident participation on health is unclear. The research was based on a “passive view” [33] that considers participation to be a goal in and of itself, rather than considering participation as a means to fulfill the goal of changing health behavior and improving physical and psychological health.

Minimizing attrition is crucial in intervention studies because follow-up loss could affect the strength of a study’s findings. The attrition rate of this study is relatively low compared to the previous studies of older adults. Strategies used to minimize attrition includes the collection of detailed contact information, encouragement of participation with postcards and calls, incentives for study participations, and enhancement of social support. As program factors, the exercise session added an element of fun by using rhythmic music selected by the participants, and the intensity of the exercises was adjusted based on one’s exercise capacity. Furthermore, connections were made between the participants and the medical doctors practicing in the participants’ area of residence for direct treatment and consultation to enable continuous treatment and monitoring after the intervention, rather than concluding with a single education session. This design was intended to prevent loss to follow-ups. Our findings suggest that consistent and ongoing engagement in the program has induced positive and statistically significant changes in outcome variables in the experimental group as compared to the control group.

This study has several limitations. First, a non-equivalent control group study was used because it was difficult to conduct randomization for ethical or practical reasons. Participants were selected from the waiting list of the home-visiting nursing service of a public health center. Given that the service is designed for a vulnerable population as public health efforts at the national level, the experimental group was created first according to the waiting list order. Even though homogeneity tests revealed no significant differences according to the group’s general characteristics and outcome variables pre-intervention, further randomized studies need to be considered to control for the effects of unexpected confounding variables on the outcome variables, or selection bias. Second, most of the participants in this study were women. The incidence, determinant, and trajectory of frailty as well as life expectancy differ by gender [34–36]. Gender-specific intervention strategies need to be developed, and future studies should oversample men to ensure parity and ability for gender specific analyses. Additionally, this study involved a 12-week (once per week for 12 weeks) intervention, and a follow-up measurement 12 weeks after the intervention. Participants may show positive changes in certain outcome variables. However, the intervention’s duration and intensity may not have been sufficient to modify physical functions, such as handgrip strength and frailty. Future studies with longer interventions and higher frequent measurements are required to draw greater empirical conclusions regarding the outcome variables.

## Conclusions

Given that Korea is rapidly aging, the proportion of frail and older adults living alone will inevitably increase. Therefore, it is crucial to assist older adults in living safely and independently in the community for as long as possible. This study showed that nurse-led multicomponent intervention was effective for prefrail and frail older adults living alone in Korea, presenting the possibility of proactive management regarding their health and leading their daily life. Therefore, a strategy for disseminating widely sustainable nurse-led multicomponent interventions should be developed for community-dwelling frail elderly who live alone.

## Data Availability

The datasets used and/or analyzed during the current study are available from the corresponding author on reasonable request.

## Abbreviations

SES: socioeconomic status
TUG: Timed Up and Go Test
GDSSF-K: Geriatric Depression Scale-Short Form Korean Version
